# Experimental Analysis of Porosity and Permeability in Pressed Paper

**DOI:** 10.3390/mi7030048

**Published:** 2016-03-14

**Authors:** Juhwan Park, Joong Ho Shin, Je-Kyun Park

**Affiliations:** Department of Bio and Brain Engineering, Korea Advanced Institute of Science and Technology (KAIST), 291 Daehak-ro, Yuseong-gu, Daejeon 34141, Republic of Korea; juhwanpark@kaist.ac.kr (J.P.); stvshn32@kaist.ac.kr (J.H.S.)

**Keywords:** paper-based microfluidics, porosity, permeability, pressed paper

## Abstract

In this paper, we report an analysis of pressed paper in terms of porosity and permeability. Previously, we reported a pressed paper that exhibits decreased porosity and permeability. Additionally, its applications into programmed sample delivery as well as flow rate control were reported. However, there is a need for a theoretical analysis of pressed paper in terms of porosity and permeability for a more precise design principle and its applications because porosity and permeability are important factors in determining fluidic behavior. Here, we propose a theoretical model for analyzing decreased porosity and permeability in pressed paper. Porosity and permeability of pressed paper were quantitatively calculated using experimental results with a theoretical model. Furthermore, based on the analyzed results of porosity and permeability in pressed paper, a porosity–permeability relationship was investigated.

## 1. Introduction

Paper has been widely used as a detection platform because of its advantages, including low cost, wide availability, and capillary driven simple operation principle [1,2,3,4]. Recently, a number of microfluidic paper-based analytical devices (μPADs) have been developed by realizing patterned paper in various forms to perform biological assays [5,6,7,8,9,10]. Although μPADs confined fluid flow along capillary channel by forming hydrophobic barriers on paper, they did not control fluid flow along capillary channels. However, there is a need for controlling the fluid flow along capillary channels in order to develop μPADs that can handle complicated reaction steps. To realize complicated reaction steps, many methods for controlling fluid flow on paper have been reported, including surfactant based fluidic diode valves [11], geometry modification of fluidic channel [12,13], dissolvable barriers for time delays [14], and paraffin infused paper [15]. However, the above-mentioned methods require complicated fabrication processes, such as chemical treatment and spatially limited geometry modification.

As a simple method for controlling fluid flow on paper, we recently reported on pressed paper and its application to flow rate control and programmed sample delivery [16]. Controlling fluid flow was easily demonstrated by collapsing the porous network. On the other hand, the physical collapse of porous network usually occurs in a calendering process, which is conventionally used to finalize paper manufacturing to provide a smooth and shiny surface. Moreover, the effects of the calendering process have been studied regarding morphology of fibers and surface properties of paper [17,18,19]. However, in this paper, capillary flow was controlled by collapsing the porous network, which results in decreased porosity and permeability. In general, for porous materials, porosity and permeability are important factors in determining characteristic of fluidic behavior [20,21]. To date, many theoretical studies for capillary flow in various paper-based microfluidic devices, including two-dimensional paper networks [22], wax-printed paper [23], and porous membrane in complex shape [24], have been reported in order to understand fluid dynamics. Therefore, a theoretical and experimental analysis for decreased porosity and permeability in pressed paper is required to understand controlled capillary flow in pressed paper. Moreover, an analysis of decreased porosity and permeability is also required for a more precise design principle and its applications. Additionally, permeability is usually represented as a function of porosity in porous material such that porosity–permeability relationships have been studied with many theoretical models [25,26]. In this sense, an analysis of decreased porosity and permeability in pressed paper would be helpful in understanding porosity–permeability relationships in porous material.

Here, we present a theoretical and quantitative analysis of porosity and permeability in pressed paper. Pressed paper exhibits decreased porosity and permeability, which results in a decreased flow rate as well as delayed fluid flow. To understand these phenomena in detail, we constructed a theoretical model for analyzing porosity and permeability in pressed paper. Additionally, for a precise design of paper-based microfluidic devices that include pressed regions, the porosity and permeability of pressed paper were calculated quantitatively. Finally, based on the analysis of porosity and permeability in pressed paper, a porosity–permeability relationship was investigated.

## 2. Experimental

### 2.1. Materials

Tartrazine (yellow dye) and erioglaucine (blue dye) were purchased from Sigma-Aldrich (St. Louis, MO, USA). An absorbent pad (grade 222) and sample pad (grade 319) were purchased from Ahlstrom (Helsinki, Finland). A glass fiber pad (GFCP103000) was purchased from Millipore (Billerica, MA, USA). A nitrocellulose (NC) membrane (CNPC-SS12, 15 μm) was purchased from Advanced Microdevices (Ambala Cantt, India).

### 2.2. Thickness Measurement of Pressed Paper

A NC membrane was cut into strips (width = 4 mm) using a knife. After an acrylic plate was placed on a desirable position of the NC membrane strip, it was partially pressed with a hand press machine (SWP-HP180-120S; SamWoo, Siheung, Korea). The amount of applied pressure was measured by monitoring an indicator connected to load cell (CLS-1 T; Curiosity Technology, Paju, Korea), which is placed on the hand press machine. The side view of pressed NC membrane strip was captured with a scanning electron microscope (SEM). The thickness of the pressed region in the pressed NC membrane strip was analyzed with ImageJ software (http://rsbweb.nih.gov, NIH, Bethesda, MD, USA).

### 2.3. Measurement of Decreased Flow Rate in Pressed Paper for Analyzing Decreased Permeability

A NC membrane was cut into the desired shape via laser cutting (laser cutter C40-60W; Coryart, Anyang, Korea). To compare flow rates along different paper fluidic channels, a channel partition was developed to divide the NC membrane into two different channels. One channel was pressed with a hand press machine with different amounts of applied pressure, and the other channel was unpressed. A 1-mM tartrazine solution (yellow dye) and a 1-mM erioglaucine solution (blue dye) were supplied into each channel along the glass fiber and sample pad. Fluid flow along the NC membrane continued until each flow reached equilibrium by placing an absorbent pad at the end of the NC membrane. After fluid flow along both channels reached equilibrium, wetted portions of each dye solution in a detection region were analyzed with ImageJ software.

### 2.4. Porosity Measurement of Unpressed Paper

Porosity of an unpressed NC membrane was experimentally measured. Void volume in the NC membrane strip (5 mm × 30 mm) was obtained by measuring the absorbed volume of distilled water. Then, porosity of the unpressed NC membrane was calculated by dividing the absorbed volume of distilled water by the whole volume of the unpressed NC membrane strip.

### 2.5. Darcy’s Law

When infinite sample volume is supplied at source, and the capacity of absorbent pad is infinite, the flow rate along the paper fluidic channel at fully-wetted flow can be described by Darcy’s law, as per the following equation:

(1) $$Q=-\frac{\kappa WH}{\mu L}\Delta P$$

where *Q* is the volumetric flow rate, κ is the permeability of paper, *W* is the width of paper, *H* is the thickness of paper, μ is the viscosity of fluid, *L* is the length of paper, and Δ*P* is the pressure difference along the paper fluidic channel.

## 3. Results and Discussion

### 3.1. Pressed Paper

**Figure 1:**
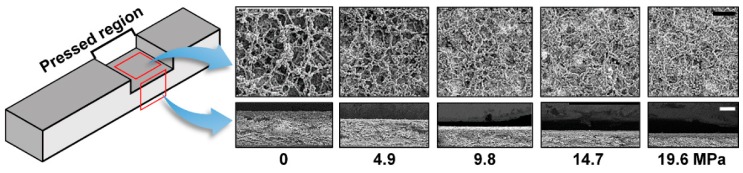
Schematic of pressed paper. Top view (scale bar = 20 μm) and side view (scale bar = 50 μm) of scanning electron microscope (SEM) images in pressed region.

The porous network of paper collapses when it is exposed to applied pressure. As shown in Figure 1, the pressed region shows a decreased pore size and thickness that can be understood as a decrease in permeability according to the increasing amount of applied pressure. The collapsed porous network plays a role in fluid resistance that results in a delayed fluid flow and decreased flow rate. In the case of the wet-out process, the quantity of fluid flow that can penetrate a pressed region decreases with respect to the increasing amount of applied pressure. As the quantity of fluid flow that passes through a pressed region decreases, a wicking time for the left portion of the paper after the pressed region increases. Therefore, a delayed fluid flow occurred in the pressed paper during the wet-out process. On the other hand, during fully-wetted flow, a decreased flow rate in the pressed paper can be understood in terms of decreased permeability and thickness in Darcy’s law. Finally, flow rate along the pressed paper decreases with an increasing amount of applied pressure.

### 3.2. Porosity of Pressed Paper

**Figure 2:**
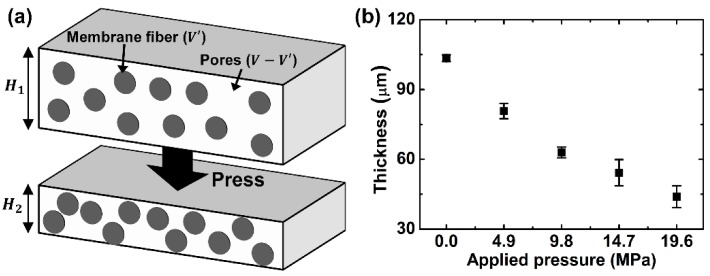
Analysis of porosity in pressed paper. (**a**) A schematic of decreased porosity in pressed paper; (**b**) Graph of decreased thickness in pressed region according to increasing amount of applied pressure.

Porosity is a measure of the void space, which is an important factor in determining the characteristics of porous materials. The porosity of paper can be calculated with the following equation:

(2) $$\text{Porosity}\;\left(p\right)=1-\frac{V^{\prime }}{V}$$

where *V′* is the volume of fiber and *V* is the total volume of paper material. When a paper is exposed to applied pressure, pores are collapsed, resulting in a decreased thickness and pore size. The collapsed structure of paper does not recover over time. This can be explained by the fact that the volume of pores (*V − V′*) decreases while the volume of fiber (*V′*) does not decrease, as shown in Figure 2a. Accordingly, the volume ratio of fibers in the pressed paper can be obtained by dividing the volume of fiber (*V′*) by the entire volume of the pressed paper (*V·*(*H*_2_*/H*_1_)). After that, the porosity of the pressed paper can be calculated by subtracting the volume ratio of fibers ((*V′/V)·*(*H*_1_*/H*_2_)) in the pressed paper. Finally, the porosity of the pressed paper can be calculated in terms of the unpressed paper’s porosity (*p*) and the decreased ratio of thickness in the pressed paper (*H*_1_*/H*_2_), as per the following equation:

(3) $$\text{Porosity of pressed paper}\;\left(p^{\prime }\right)=1-\frac{H_{1}}{H_{2}}\left(1-p\right)$$

where *H*_1_ is the thickness of the unpressed paper and *H*_2_ is the thickness of the pressed paper. The thickness of the pressed region decreased according to the increase in applied pressure (Figure 2b). Because a decrease in thickness of the pressed region was almost saturated after 19.6 MPa, we determined a range of measurements from 0 to 19.6 MPa. Based on an average value of thickness in the pressed paper, we calculated the porosity of the pressed paper quantitatively using Equation (3). Porosity of the unpressed NC membrane was experimentally obtained by measuring the absorbed volume of distilled water into a NC membrane. It was found to be 0.76 ± 0.03 (*n* = 10). Accordingly, the calculated porosities of pressed papers were 0.69, 0.60, 0.54 and 0.44, respectively.

### 3.3. Flow Rate along Pressed Paper

**Figure 3:**
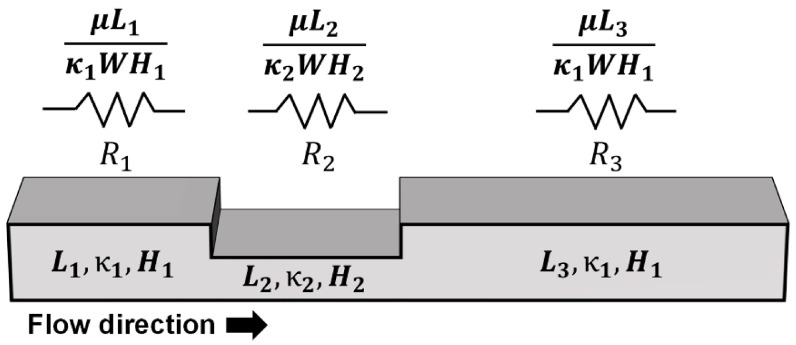
Pressed paper and its comparison with electrical circuit for calculating flow rate.

By using an analogy of an electric circuit, *Q* can be considered as current, *ΔP* can be considered as voltage, and μ*L/*κ*WH* can be considered as resistance in Darcy’s law (Equation (1)). Based on this analogy, the paper strip that includes many different segments can be considered a serial connection of different resistances [22]. Therefore, the flow rate along the pressed paper can be calculated by dividing *ΔP* by the sum of three different resistances (μ*L*_1_*/*κ_1_*WH*_1_ *+* μ*L*_2_*/*κ_2_*WH*_2_ *+* μ*L*_3_*/*κ_1_*WH*_1_) (Figure 3). Because the overall length of pressed paper *L* can be represented as the sum of the length of each segment (*L*_1_* + L*_2_* + L*_3_)*,* the flow rate in pressed paper can be calculated with the following equation:

(4) $$Q=-\frac{W\Delta P}{\mu \left(\frac{L}{\kappa_{1}H_{1}}+L_{2}\left(\frac{1}{\kappa_{2}H_{2}}-\frac{1}{\kappa_{1}H_{1}}\right)\right)}$$

Based on Equation (4), it is supposed that the flow rate along the pressed paper is affected by the length, the permeability, and the thickness of the pressed region. Decreased flow rate along the pressed paper can be explained in terms of decreased permeability and the thickness of the pressed region [16]. Although we already demonstrated a decreased flow rate in pressed paper according to different amounts of pressure, it is supposed that flow rate can be controlled by differentiating the length of the pressed region. Moreover, on the basis of Equation (4), we can suppose that a decreased flow rate along the pressed region would not be affected by the position of the pressed region. It is worth noting that the amount of pressure and the length of the pressed region is a control factor of flow rate control, regardless of the position of the pressed region. Furthermore, the flow rate of the pressed paper that includes multiple pressed regions can be easily analyzed by the same analogy as above.

### 3.4. Permeability of Pressed Paper

**Figure 4:**
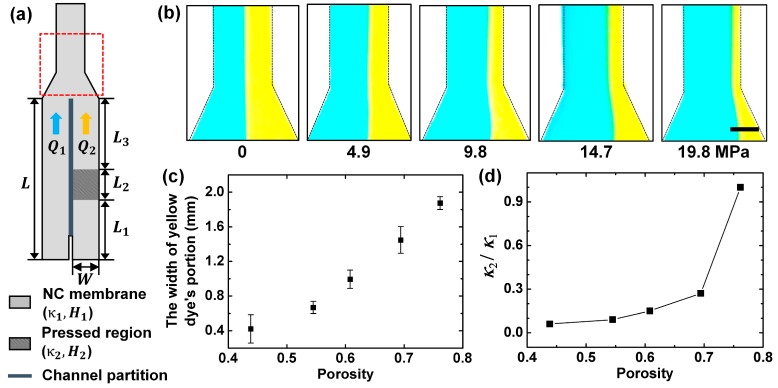
Measurement of decreased permeability in pressed paper. (**a**) Experimental setup for comparing flow rate along two different channels; (**b**) Experimental results of flow rate comparison. Images are captured at red dotted box in panel **a** (scale bar = 2 mm); (**c**) Graph of decreased width of yellow dye’s portion according to the porosity of pressed paper; (**d**) A porosity–permeability relationship in pressed NC membrane.

Generally, the permeability of paper can be calculated by utilizing Darcy’s law. To calculate the decreased amount of permeability, the decreased flow rate in the pressed paper (Equation (4)) was compared with the flow rate in the unpressed paper (−κ*_1_WH*_1_Δ*P/*μ*L*). Therefore, we designed a pressed paper-based microfluidic device, as shown in Figure 4a. By comparing the flow rate along two different channels, where one includes a pressed region while the other does not, κ_2_/κ_1_ can be calculated with Equation (5) assuming that the pressure difference (Δ*P*) along each channel and the viscosity (μ) of each solution are almost the same.

(5) $$\frac{\kappa_{2}}{\kappa_{1}}=\frac{L_{2}\frac{H_{1}}{H_{2}}}{L\left(\frac{Q_{1}}{Q_{2}}-1\right)+L_{2}}$$

To calculate κ*_2_/*κ*_1_* quantitatively, *Q_1_/Q_2_* and *H_1_/H_2_* is required in Equation (5). *Q_1_/Q_2_* was easily obtained by measuring the width of each dye’s portion at different amounts of pressure because the flow rate along each channel can be represented in terms of the width of each dye’s portion (Figure 4b) [13]. Therefore, we measured the width of the yellow dye’s portion at the detection region after the flow along both channels reached equilibrium. The width of the yellow dye’s portion decreases with an increasing amount of pressure, which means a decrease in flow rate along a channel that includes a pressed region. The width of the yellow dye’s portion increased with the increasing porosity of the pressed paper (Figure 4c). Finally, by analyzing the average value of *Q_1_/Q_2_* obtained from Figure 4c and the average value of *H_1_/H_2_* obtained from Figure 2b, κ*_2_/*κ*_1_* was calculated quantitatively (*L* = 2.5 cm and *L_2_* = 0.5 cm). Each value of κ*_2_/*κ*_1_* in the pressed paper was 0.06, 0.09, 0.15 and 0.27, respectively. It is expected to be useful for designing a delayed fluid flow as well as a decreased flow rate in pressed paper. Although we represented a ratio of decreased permeability instead of a real value of permeability due to the limited experimental setup in the laboratory, the real value of permeability in pressed paper can easily be calculated by measuring the permeability of unpressed paper using a reported method [27].

Furthermore, we studied a porosity–permeability relationship by comparing a decreased porosity and permeability in pressed paper. Permeability was dramatically decreased with respect to decreasing porosity (Figure 4d). More detailed studies for a porosity–permeability relationship can be achieved by measuring porosity and permeability at a dynamic range of applied pressure for the pressed region. Additionally, many kinds of NC membranes could be adjusted for a more practical study. Although it is clear that fluid cannot easily wick into paper when porosity is low, a quantitative analysis of decreased permeability with respect to decreasing porosity should be studied. In this sense, many theoretical models for a porosity–permeability relationship in porous materials have been reported [25,26]. However, these are based on simulation results because experimental results regarding porosity and permeability are difficult to obtain. On the other hand, both porosity and permeability are controllable by pressing paper in contrast to other chemical methods for the modification of paper. Therefore, it is worth noting that a porosity–permeability study can be performed on one kind of paper without the fabrication of a new paper that exhibits a different porosity and permeability. Accordingly, pressed paper can be a useful experimental tool for studying porosity and permeability. Furthermore, a porosity–permeability relationship in pressed paper is expected to be widely useful in the design of paper-based microfluidic devices that include pressed regions.

## 4. Conclusions

In this work, we reported an experimental analysis regarding porosity and permeability in pressed paper. After constructing a theoretical model for analyzing pressed paper, we calculated decreased porosity and permeability quantitatively. Based on the experimental results, which consisted of thickness changes in a pressed region and a comparison of decreased flow rates, porosity and permeability at each applied pressure were obtained. Because porosity and permeability are important factors in determining fluidic behavior in paper, it would be useful to design a microfluidic device for pressed paper. In addition, it would be also helpful to understand fluidic behavior in pressed paper that exhibits a decreased flow rate as well as a delayed fluid flow. Furthermore, we proposed the possibility of pressed paper being utilized as an experimental tool for studying porosity–permeability relationships because both porosity and permeability were both controllable by pressing the paper.
